# Dairy and Headaches: What is the Connection?

**DOI:** 10.1007/s11916-024-01303-w

**Published:** 2024-07-27

**Authors:** Merve Ceren Akgör, Esme Ekizoğlu, Aynur Özge

**Affiliations:** 1 Department of Neurology, University of Health Sciences Ankara Atatürk Sanatoryum Training and Research Hospital, Ankara, Turkey; 2 Neuroscience and Neurotechnology Center of Excellence (NÖROM), Gazi University, Ankara, Turkey; 3 Istanbul Faculty of Medicine, Department of Neurology, Istanbul University, Istanbul, Turkey; 4 Department of Neurology, School of Medicine, Mersin University, Mersin, Turkey

**Keywords:** Migraine, Triggers, Food triggers, Dairy products, Tyramine, Histamine, Lactose intolerance, Headache management, Dietary interventions

## Abstract

**Purpose of Reviews:**

Headaches represent a prevalent and burdensome health condition, affecting individuals of all ages worldwide. While dietary factors have been implicated in headache pathophysiology, the association between dairy consumption and headaches remains controversial and inadequately understood. This comprehensive review systematically examines the existing literature to elucidate the relationship between dairy intake and headaches, addressing methodological challenges, potential biases, and gaps in the current knowledge.

**Recent Findings:**

A thorough search of electronic databases identified relevant observational studies, clinical trials, and mechanistic investigations exploring the impact of dairy consumption on headache incidence, frequency, severity, and duration. Methodological considerations, including study design, measurement of exposure and outcome variables, confounding factors, and sources of bias, were critically evaluated to assess the strength of evidence and validity of findings. Despite heterogeneity across studies, emerging evidence suggests a complex and multifaceted relationship between dairy intake and headaches, influenced by individual characteristics, dietary patterns, headache subtype, and study context. While some studies report a positive association between dairy consumption and headaches, others indicate no significant effect or potential therapeutic benefits of dairy restriction. Mechanistic insights suggest plausible biological mechanisms, including neuroinflammatory pathways, neurotransmitter modulation, vascular effects, and gut-brain interactions, which may mediate the observed associations.

**Summary:**

Future research directions encompass longitudinal studies, mechanistic investigations, stratified analyses, randomized controlled trials, and exploration of the gut microbiota to further elucidate the underlying mechanisms and inform evidence-based dietary recommendations for headache management. This integrative review underscores the importance of interdisciplinary collaboration and personalized approaches to address the complex interplay between diet, headaches, and overall health.

## Introduction

Headache is a common and often debilitating health problem that affects a significant proportion of the world’s population. These painful episodes vary in intensity, duration, and triggers, making the identification of potential causative factors a complex and essential endeavor. While dietary patterns have long been suspected to play a role in headache etiology, the relationship between dairy consumption and headaches remains a topic of considerable interest and debate. Understanding the connection between dairy and headache is important for several reasons. First, dairy products are a staple in many people's diets worldwide, and the potential health effects of their consumption, including their role in headaches, merit through investigation. Secondly, identifying specific dairy components that may act as headache triggers is crucial for personalized dietary recommendations, which may offer relief to those who experience dairy-related headaches.

This review aims to provide a comprehensive analysis of the current state of knowledge on this field, shedding light on the complex relationship between dairy consumption and headaches. To this end, we also aimed to evaluate the potential mechanisms by which dairy products may be linked to headaches, such as the presence of compounds like tyramine and histamine which have been proposed as triggering compounds in dairy products [1]. Additionally, the prevalence and impact of lactose intolerance, a condition that can complicate the relationship between dairy products and headaches will be discussed. It is worth noting that lactose intolerance is more common than is often realized and may be an overlooked factor in the headaches experienced by dairy consumers [2, 3].

The complex relationship between migraines and dairy products will also be a focus point of discussion in this article. Unpacking this complexity is crucial for a better understanding of the issue.

In sum, this review article sets out to dissect the complex interplay between dairy consumption and headache. Through an evidence-based exploration of this relationship, a broader understanding of dietary influences on headache etiology will be provided, ultimately offering insight and guidance to individuals seeking relief from dairy-related headaches will be provided.

## Dairy Consumption and Headache: An Overview

Migraine and food triggers have been the subject of investigation for many years. The foods commonly blamed as triggers are alcohol, chocolate, caffeine, monosodium glutamate, and dairy products such as aged cheese. However, the relevant mechanisms have not been fully understood yet. Dairy products are among the most widely consumed foods by humans throughout their lifetime, starting from birth, due to their rich content of various micronutrients such as calcium, phosphorus, magnesium, vitamin B12, riboflavin, and vitamin D.

It is difficult to interpret the studies investigating the relationship between dairy products and migraine, due to the heterogeneity of dairy products. However, a noteworthy observation in the literature is that processed dairy products are more often implicated as triggers compared to unprocessed dairy products. In particular, aged cheese with a high fat content has been found to trigger migraine attacks more often than fresh milk and cheese. This is thought to be due to the higher levels of tyramine, a vasoactive compound, in aged fatty cheeses [4].

Dairy products such as aged cheese and ice cream, have been identified as potent triggers for migraine patients [5].

A recent study revealed that foods such as milk, aged cheese, full-fat cheese, and ice cream were more frequently associated with chronic migraine in comparison to episodic migraine [6]. This relationship in question varies also among products. Cheese was identified as a trigger in 8.5% of the cases, while milk had a trigger incidence of 2.5% in Brazil [7]. Another study from Belgium reported that ice cream was twice as likely to cause headaches as milk, with an incidence of 4.6% [8]. Furthermore, all types of cheese were found to be highest- ranking food triggers compared to other foods according to the findings of a study conducted in Turkiye, that evaluated food triggers in secondary vertigo attacks associated with migraine [9]. This association has also been shown to affect patients’ dairy preferences. A study of women with migraine reported a decrease in the consumption of dairy products, aged cheese, sour cream, and milk. This phenomenon has been interpreted as a behavioral pattern of avoiding triggers in people with migraines [10]. While dairy products have been implicated as migraine triggers in many studies, some reported that adequate consumption of low-fat dairy products may reduce migraine attacks in children and adolescents [2].

We have noticed two predominant views on this issue, based on the reports in the literature. The first viewpoint suggests that certain dairy products, such as processed cheese, whole milk, cream, and ice cream are often reported as headache triggers in patients with migraine because of the food components they contain, indicating a potential intolerance in these individuals. These foods should therefore be avoided by patients with migraine. Based on previous studies, low-fat milk or dairy products with lower tyramine content are considered safer alternatives for migraine patients. The second view is that certain nutrients found in different food sources may have a protective effect against migraines. Examples of these nutrients include magnesium, calcium, phosphorus, and vitamin D, all of which are found in dairy products. It has therefore been suggested that dairy products may reduce migraine attacks because they are rich in these nutrients [11].

## Lactose Intolerance and Headaches

Lactose and food intolerance manifest across a broad spectrum of symptoms, including bloating, abdominal discomfort, alternating episodes of diarrhea and constipation, severe headaches, fatigue and cognitive impairment such as difficulties concentration [12]. Despite many indications that patients with irritable bowel syndrome often report lactose intolerance, there is no conclusive evidence of an objective link between lactose intolerance and IBS. Further research is needed to establish this link. Migraine and IBS are known to be triggered by various dietary factors and people with migraine have a higher prevalence of IBS than the general population [13]. A survey study found that dairy products in milk, yogurt, ice cream, and cheese were playing as triggers in individuals with migraine, medication overuse headaches (MOH), and IBS [5].

Although migraine has been reported to be more frequently seen in people with lactose intolerance than in the general population, studies investigating the frequency of lactose intolerance and migraine are lacking, and further research is needed in this area. There is also a need for more studies on the coexistence of IBS and lactose intolerance. In addition, the possibility of confusion or coexistence of lactose intolerance and IBS has been suggested in the previous reports due to the similarity of these conditions. If lactose intolerance is suspected, it is recommended to take a detailed medical history and eliminate lactose-rich products from the diet for two weeks. Lactose malabsorption can be diagnosed only if the symptoms improve following this dietary regulation [14]. Additionally, migraine patients suspected of having lactose intolerance may be advised to keep a diary and monitor the frequency and severity of their headaches over two weeks to see if there is a reduction in headache attacks while avoiding lactose-containing foods.

If the frequency and severity of headaches decrease when lactose-containing products are removed from the diet, this observation will support the hypothesis that lactose intolerance may exert its effects via the central nervous system by increasing inflammatory responses or triggering migraine attacks. However, it is not easy to establish a clear link between migraine and lactose intolerance because of the individual differences. Further clinical trials and research are needed to better understand this potential link. It is noteworthy that there is no current study in the literature that demonstrates the relationship between migraine, or any other type of headache, and lactose intolerance. We observed a reduction in the frequency and severity of headaches in our patients suspected of migraines, lactose intolerance, or irritable bowel syndrome in whom lactose-containing foods were eliminated for 3–4 weeks.

## Migraine and Dairy: A Complex Relationship

The exact mechanisms by which foods trigger headaches are not yet fully understood, so the possible mechanisms by which dairy products, including milk, act as triggers remain uncertain. Several potential mechanisms have been considered in previous reports. One of these mechanisms is thought to be the impact of tyramine. Tyramine is an endogenous biogenic monoamine originating from the amino acid tyrosine. The potential link between biogenic monoamines such as tyramine and migraine attacks has been postulated based on their association with milk and dairy products [15].

Tyramine is present in certain cheeses, dairy products, and certain alcoholic beverages. Similar to 5- hydroxytryptamine, dopamine, and epinephrine, it undergoes oxidative deamination in the body, facilitated by the enzyme monoamine oxidase. Tyramine induces the release of endogenous aromatic amines such as noradrenaline from sympathetic nerves [16]. Blackwell and Mabbitt observed that patients taking monoamine oxidase inhibitors developed sometimes severe headaches following the consumption of cheeses and dairy products. It has been shown that the occurrence of headaches is related to the inhibition of monoamine oxidase, which is responsible for the absorption of tyramine and its breakdown in the intestine [17]. In an earlier randomized controlled trial (RCT) conducted in 1971, migraine patients had a significantly greater increase in free plasma tyramine and a decrease in conjugated tyramine compared with controls. This finding raised the question of whether there is an enzyme deficiency in tyramine conjugation in migraine [17]. Recent studies over the past 20 years have suggested that biogenic amines like tyramine, and tryptamine, contribute to the pathogenesis of migraine [18].

**Fig. 1:**
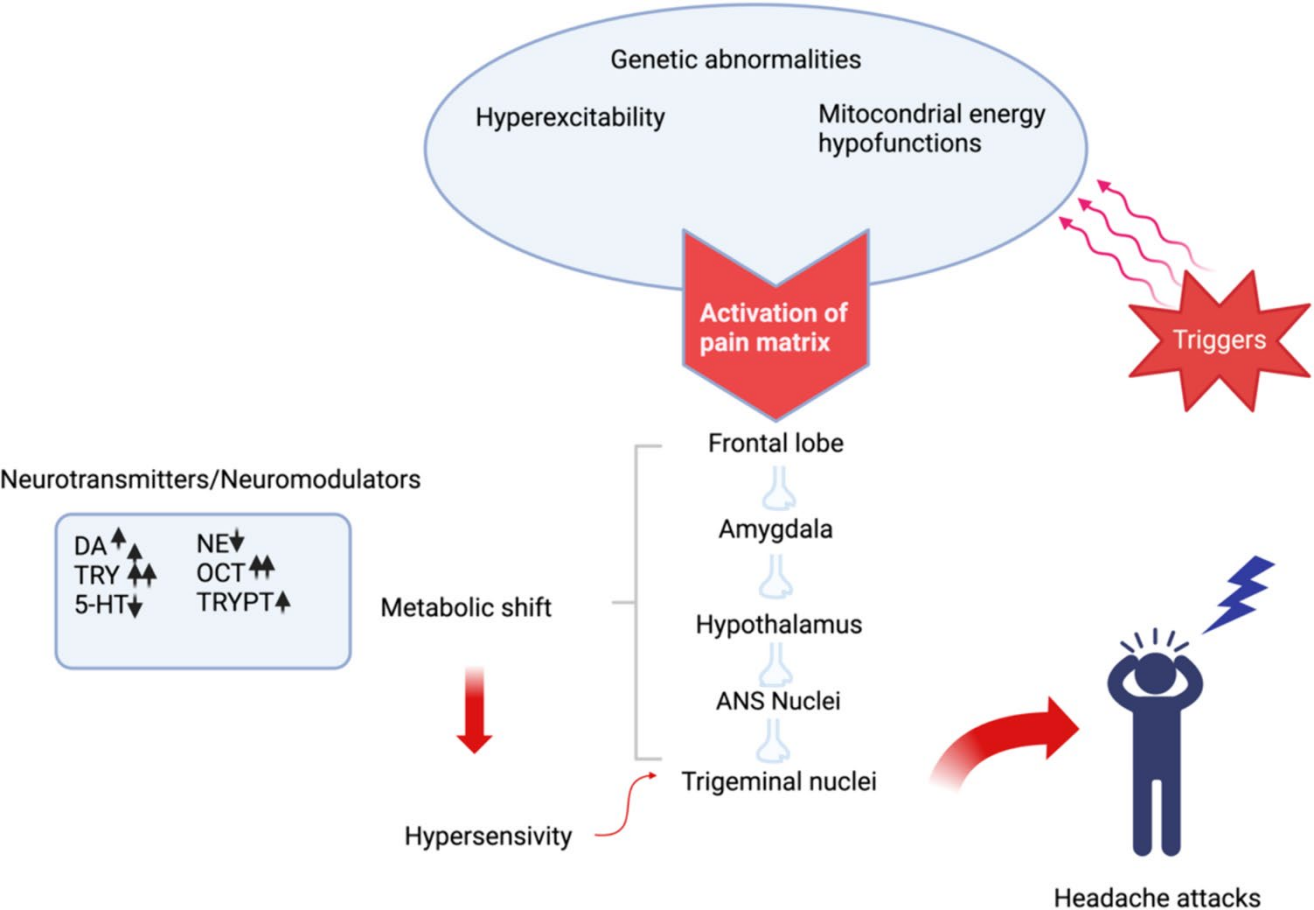
Schematic representation of neurotransmitters and enigmatic amines in the pathophysiology of migraine. (ANS: Antinociceptive system, DA: Dopamine, NE: Norepinephrine 5-HT: Serotonin TRY: Tyrosine TRYPT: Tryptophan OCT: Octopamine), created with Biorender

Abnormalities in tyrosine-related metabolites have been demonstrated in chronic migraine patients. Although the mechanism is not fully understood, one hypothesis is that abnormalities in tyrosine metabolites activate the pain matrix [19]. The pathological interplay of reduced energy reserves and altered tyrosine metabolism, including elevated levels of neuromodulators and an imbalance of neurotransmitters in platelets and plasma, observed in individuals with migraines, might reflect similar biochemical irregularities within the synaptic gaps of the pain network, forming the pathological biochemical changes underlying migraine attacks (Fig. 1) [19].

In a study exploring tyrosine metabolism in the pathogenesis of chronic migraine, the plasma levels of dopamine and noradrenaline were several times higher in the patients with chronic migraine in comparison to the control group [20]. Food additives may also play a role in the occurrence of headaches. Whipping cream and heavy cream are mainly used in ready-to-eat products and contain numerous food additives such as emulsifying salts, potassium sorbate, carrageenan, and others. In a recent study investigating the role of food triggers in migraine and MOH, dairy products, particularly those found in processed foods containing additives such as carrageenan and calcium isosorbate, were shown to be potential headache triggers. Food additives and preservatives as well, had a significant inducing effect on headache attacks as well as specific foods. In the same study, foods such as milk, cream cheese, whipped cream, cream cheese, and milk powder were found to be common food triggers for both migraine and IBS, and patients with MOH and IBS avoided consuming milk, cream cheese, and cream [1, 5].

**Fig. 2:**
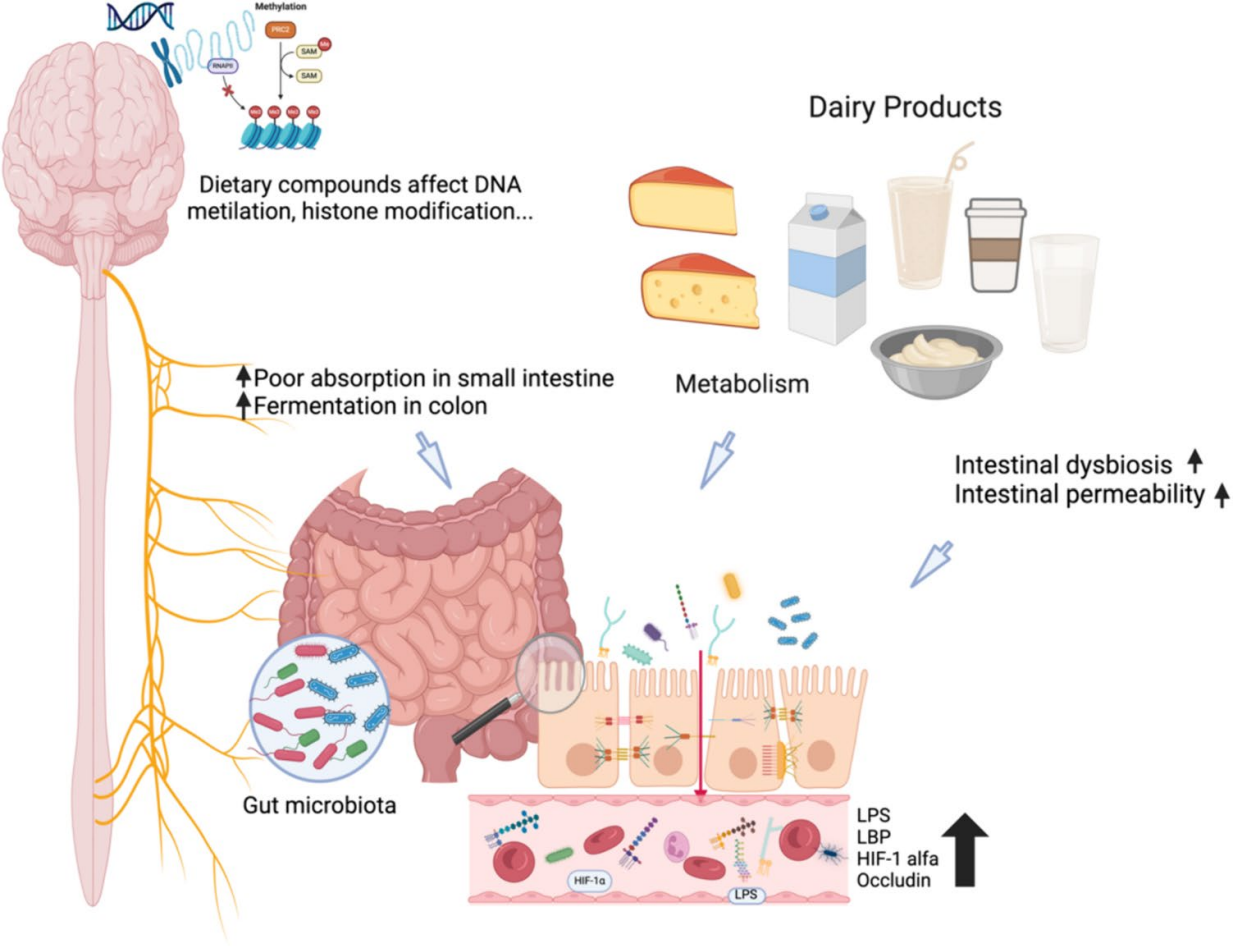
The potential contribution of increased intestinal permeability to neuroinflammation: metabolism of dairy products along with the increased fermentation in the colon, poor absorption in the small intestine, intestinal dysbiosis, and permeability individuals leading to the production of particular microbiota profiles that are more prone to trigger headaches., in addition to the epigenetic changes triggered by diet [13, 21]. Figure 2 created with Biorender

Another question that remains to be answered is whether many migraine sufferers have a food-based inflammation at varying severities. Current knowledge about the association between migraine and dairy favors a complex relationship with various aspects among these two entities. Neurogenic inflammation is well known to have a role in the pathophysiology of both migraine and IBS. Increased permeability of the intestinal barrier and the subsequent inflammation have been linked to increased occurrence of migraine. Leaky gut syndrome has been more frequently observed in chronic migraine patients with medication overuse and its pathogenesis has been demonstrated to involve increased inflammatory markers such as LPS, VE-Cadherin, HMGB-1and HIF-1alpha (Fig. 2) [21].) It seems reasonable to suggest that a wide spectrum of food-based inflammation may precipitate migraine attacks.

Dietary compounds can affect all major elements of the cellular epigenetic profile: DNA methylation, histone modification, and the effects of non-coding RNAs (ncRNAs), resulting in changes in transcription and/or translation [22]. It has been hypothesized that this phenomenon could be explained by allergic reactions to dairy proteins. These allergic reactions activate the immune system, leading to neuroinflammation through antibodies such as IgE and IgG, which may result in cerebral vasodilation and trigger migraine headaches. Another hypothesis is a possible involvement of food intolerance associated with the brain-gut axis [23].

The neurotransmitter-based hypothesis is as follows; dairy products such as ice cream, and yogurt which contain high levels of tyrosine are also rich in histamine and several authors suggested that histamine-rich foods may trigger migraines. Histamine intolerance is a condition characterized by the imbalance in histamine homeostasis, primarily due to impaired intestinal degradation of this amine often due to a deficiency of the enzyme diamine oxidase (DAO). Headache is a prominent symptom among the many histamine-related manifestations. Current clinical strategies for the management of symptoms associated with this disorder involve the exclusion of histamine-rich foods or other bioactive amines, coupled with exogenous DAO supplementation [24]. However, the lack of efficacy of histamine antagonists in preventing headache attacks suggests that histamine alone may not be the only substance involved in the mechanism [25].

Foods rich in tyrosine are also considered dopaminergic foods since tyrosine is an essential amino acid converted to dopamine. Dairy products such as milk, cheese, and yogurt are among the foods rich in the amino acid tyrosine. The relationship between dietary habits and inflammation has therefore been the subject of long-standing research. Furthermore, the consumption of high-fat cheeses and cream sauces has been shown to increase inflammatory parameters in humans, especially IL-6 [26]. A survey study mentioned earlier showed that the food triggers for MOH patients were mainly dopaminergic foods, including milk, cream, and cream cheese [5].

## Methodological Challenges

While the existing literature provides valuable insights into the relationship between dairy consumption and headaches, several methodological challenges warrant consideration. One notable challenge is the heterogeneity of study designs and methodologies across different investigations. Variations in study populations, dietary assessments, headache diagnoses, and outcome measures pose difficulties in synthesizing findings and drawing conclusive interpretations. Standardization of research methodologies, including validated tools for assessing dietary intake and headache frequency, would enhance the comparability and reliability of study results. Moreover, the inherent complexity of dietary patterns and the multitude of potential confounding factors present challenges in isolating the specific effects of dairy consumption on headache occurrence. Addressing these confounders through robust study designs, including longitudinal cohort studies and controlled dietary interventions, is crucial for elucidating causal relationships. Another methodological challenge pertains to the retrospective nature of many studies investigating dietary triggers for headaches. Recall bias and inaccuracies in self-reported dietary data may introduce errors and compromise the validity of associations observed. Prospective studies with rigorous dietary assessments and long-term follow-up are needed to provide more reliable evidence on the temporal relationship between dairy consumption and headache outcomes. Furthermore, the lack of standardized diagnostic criteria for lactose intolerance and headache disorders poses challenges in accurately characterizing these conditions and their interplay. Clear diagnostic criteria and objective measures of lactose intolerance, such as lactose breath tests or genetic testing, would strengthen the validity of study findings and facilitate comparisons across studies [27, 28]. In light of these methodological challenges, future research efforts should prioritize methodological rigor and consistency to overcome limitations and advance our understanding of the complex relationship between dairy consumption and headaches. Collaborative interdisciplinary approaches, involving clinicians, epidemiologists, nutritionists, and headache specialists, are essential for designing well-controlled studies that address these challenges and provide robust evidence to inform clinical practice and dietary recommendations.

## Dairy-Free Diet and Headache Management

While it is believed that the antioxidants present in dairy products may reduce migraine headaches, on the other hand, survey studies on migraine and food triggers have identified dairy products as potent headache triggers in migraine patients. The pathophysiology in both cases has not yet been fully understood [29]. It is commonly considered that dairy and dairy products may induce low-grade inflammation in the serum and thus exacerbate secondary headaches through neuroinflammation, however, further RCTs are needed to confirm this hypothesis. Although reports in the literature suggest a common occurrence of lactose intolerance and migraine comorbidity, further detailed studies are warranted in this regard as well. Considering the brain-gut axis, when contemplating the relationship between dairy products and headaches, it can be speculated that individuals consuming these products may have microbiota profiles that are more prone to trigger headaches. Moreover, dairy products may introduce leaky gut syndrome, contributing to migraine patients’ sensitivity to triggers. Therefore, advanced studies examining food consumption diaries and microbiota profiles are needed in cases where leaky gut syndrome is suspected in headaches. Recommendations for patients in this regard may include the addition of food consumption diaries alongside headache diaries. If dairy products are considered triggers, these foods can be eliminated from the diet following the sequence below. In daily practice, many physicians observe a reduction in patients’ headache symptoms with dietary restrictions such as avoiding milk and dairy products. However, RCTs are needed to substantiate this observation.

It seems beneficial to advise migraineurs to keep food diaries to raise awareness of food triggers focusing especially on dairy products, in addition to their headache diary. During periods of increased headache frequency and frequent use of painkillers, migraineurs should eliminate packaged, processed dairy products containing food additives from their diet [30]. Cream cheese, cream, and desserts with whipped cream have also been reported as strong headache triggers and should be avoided during periods of increased headache frequency. Despite these recommendations, if headaches persist, it is advisable to remove fresh milk from the diet for about 8 weeks [31]. Furthermore a randomised controlled clinical trial has shown that dietary restriction based on IgG antibodies, including milk and dairy products can be beneficial in reducing the frequency of migraine attacks and in treatment- resistant patients [32]. It should be noted that headaches are not always expected to occur when the trigger food, including dairy products, is consumed. The individual's level of hyperexcitability at the time is crucial. The co-occurrence of many triggering factors may increase the individual's excitability, making it easier to trigger a headache with dairy products.

**Fig. 3:**
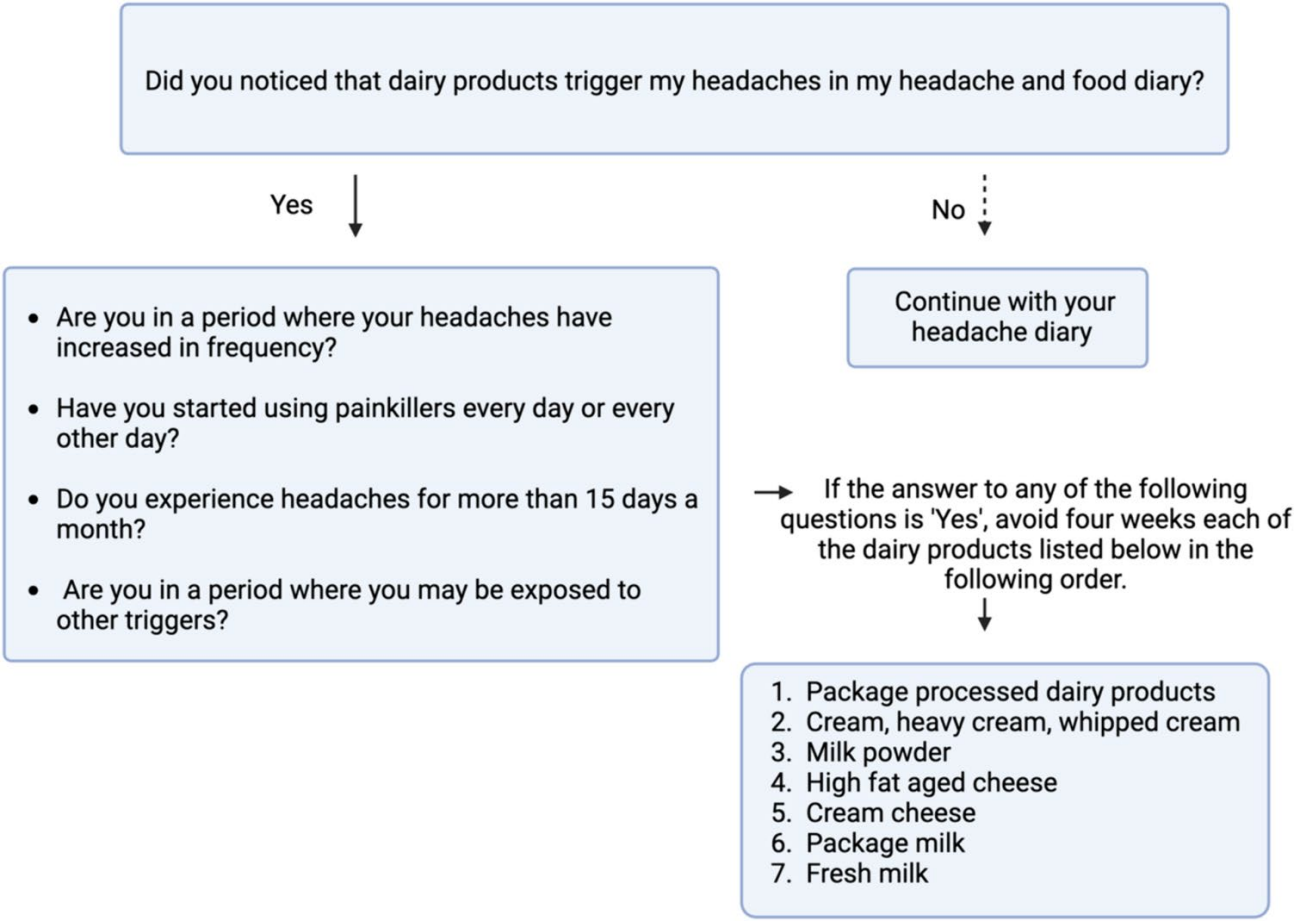
The proposed algorithm for the dairy products intake in migraine management

Temporary avoidance of dairy products is thought to be beneficial during periods of increased frequency, intensity and duration of migraine attacks, during the transition from episodic to chronic migraine, during periods of migraine prodromal symptoms, or when other triggers such as menstruation or sleep deprivation are present (Fig. 3).

## Future Directions

Many studies evaluating the relationship between headaches and dairy are self-reporting survey-based reports and there is no RCT on this subject. Therefore, more research is needed in this field. Moving forward, there are several avenues for future research to further elucidate the relationship between dairy consumption and headaches and address remaining gaps in the literature. First, longitudinal studies are needed to establish temporal relationships and assess the long-term effects of dairy intake on headache incidence, frequency, severity, and duration. By following individuals over time, researchers can better capture changes in dietary habits and headache patterns, as well as identify potential modifiers or mediators of the association. Second, mechanistic studies are warranted to investigate the biological mechanisms underlying the observed associations between dairy consumption and headaches. This may involve exploring the role of specific nutrients or bioactive compounds in dairy products, such as calcium, vitamin D, tyramine, or casein, in modulating neuroinflammatory pathways, neurotransmitter levels, vascular function, or cortical excitability implicated in headache pathophysiology. Advanced neuroimaging techniques, molecular assays, and animal models can provide valuable insights into the physiological processes involved and potential targets for intervention. Third, stratified analyses based on headache subtype (e.g., migraine, tension-type headache) and patient characteristics (e.g., age, sex, genetic predisposition) can help identify subgroups that may be more susceptible to the effects of dairy consumption on headaches. This personalized medicine approach may facilitate the development of tailored dietary recommendations or interventions to optimize headache management and improve clinical outcomes. Fourth, RCTs are needed to evaluate the efficacy of dairy restriction or supplementation as a therapeutic strategy for individuals with headaches. By comparing different dietary interventions (e.g., low-dairy diet, dairy elimination diet, dairy product supplementation) with appropriate controls, researchers can assess the impact on headache outcomes while minimizing potential confounding factors. Moreover, conducting RCTs in diverse populations and settings can enhance the generalizability of findings and inform evidence-based dietary guidelines. Fifth, studies exploring the role of gut microbiota in mediating the relationship between dairy consumption and headaches represent a promising area of investigation. Emerging evidence suggests that gut-brain interactions play a key role in migraine and other headache disorders, and dietary factors may influence gut microbial composition and function. Investigating the microbiome profiles of individuals with headaches concerning dairy intake could provide mechanistic insights and identify novel therapeutic targets for intervention.

Finally, interdisciplinary collaboration between researchers, clinicians, dietitians, and patients is essential for advancing our understanding of the complex interrelationships between diet, headaches, and overall health. By integrating diverse perspectives and expertise, we can develop holistic approaches to headache management that encompass dietary modifications, lifestyle interventions, pharmacological therapies, and psychosocial support. Furthermore, fostering patient engagement and education can empower individuals to make informed decisions about their dietary choices and optimize their health and well-being.

## Conclusion

The relationship between dairy consumption and headaches, particularly migraines, presents a complex and multifaceted interplay. While some studies suggest a link between dairy products and headache triggers, others propose potential protective effects of certain nutrients found in dairy. The mechanisms underlying these associations are not fully understood and likely involve a combination of factors, including the presence of compounds like tyramine and histamine, lactose intolerance, and inflammatory responses.

Our review highlights the importance of individualized dietary approaches for managing dairy-triggered headaches, emphasizing the need for heightened awareness among healthcare providers and patients regarding food triggers. We have discussed the potential role of lactose intolerance and its overlap with migraine, suggesting that further research is needed to elucidate this relationship.

Moreover, the possible involvement of neuroinflammation and the gut-brain axis in dairy-induced headaches warrants further investigation. Understanding these mechanisms may provide insights into personalized treatment strategies, such as dietary modifications and the elimination of specific dairy products, to improve headache management and enhance patients' quality of life. While clinical observations suggest benefits from dairy-free diets in some migraine patients, rigorous RCTs are needed to validate these findings and establish evidence-based dietary guidelines. Additionally, future research should explore the impact of dairy consumption on microbiota profiles and inflammation in migraine patients to deepen our understanding of this complex relationship.

In summary, our review underscores the need for comprehensive studies to unravel the intricate connections between dairy products and headaches, offering potential avenues for more effective treatment and prevention strategies tailored to individual patient needs. By advancing our knowledge in this field, we can strive to alleviate the burden of headache disorders and enhance the well-being of affected individuals.

## Data Availability

No datasets were generated or analyzed in the present review.

## References

[1] Worm J, Falkenberg K, Olesen J. Histamine and migraine revisited: mechanisms and possible drug targets. J Headache Pain. 2019;20(1):30. 10.1186/s10194-019-0984-1. PubMed PMID: 30909864; PubMed Central PMCID: PMCPMC6734463. 30909864 10.1186/s10194-019-0984-1 PMC6734463

[2] Ariyanfar S, Razeghi Jahromi S, Rezaeimanesh N, Togha M, Ghorbani Z, Khadem E, et al. The association between dairy intake and migraine odds among pediatrics and adolescents: A case-control study. Iran J Child Neurol. 2022;16(1):105–22. 10.22037/ijcn.v15i4.3062. PubMed PMID: 35222662; PubMed Central PMCID: PMCPMC8753001. 35222662 10.22037/ijcn.v15i4.3062 PMC8753001

[3] Mansouri M, Sharifi F, Varmaghani M, Yaghubi H, Shokri A, Moghadas-Tabrizi Y, et al. Dairy consumption in relation to primary headaches among a large population of university students: The MEPHASOUS study. Complement Ther Med. 2020;48:102269. 10.1016/j.ctim.2019.102269. PubMed PMID: 31987219. 31987219 10.1016/j.ctim.2019.102269

[4] Mollaoğlu M. Trigger factors in migraine patients. J Health Psychol. 2013;18(7):984–94. 10.1177/1359105312446773. PubMed PMID: 23104993. 23104993 10.1177/1359105312446773

[5] Ceren Akgor M, Vuralli D, Sucu DH, Gokce S, Tasdelen B, Gultekin F, et al. Distinct food triggers for migraine, medication overuse headache and irritable bowel syndrome. J Clin Med. 2023;12(20). 10.3390/jcm12206488. PubMed PMID: 37892628; PubMed Central PMCID: PMCPMC10607881. **This article forms the main framework of the present study**. 10.3390/jcm12206488 PMC10607881 37892628

[6] Fayed AI, Emam H, Abdel-Fattah AN, Shamloul RM, Elkholy TA, Yassen EM, et al. The correlation between the frequent intake of dietary migraine triggers and increased clinical features of migraine (analytical cross-sectional study from Egypt). Sci Rep. 2024;14(1):4150. 10.1038/s41598-024-54339-8. PubMed PMID: 38378909; PubMed Central PMCID: PMCPMC10879089. 38378909 10.1038/s41598-024-54339-8 PMC10879089

[7] Fukui PT, Gonçalves TR, Strabelli CG, Lucchino NM, Matos FC, Santos JP, et al. Trigger factors in migraine patients. Arq Neuropsiquiatr. 2008;66(3a):494–9. 10.1590/s0004-282x2008000400011. PubMed PMID: 18813707. 18813707 10.1590/s0004-282x2008000400011

[8] Van den Bergh V, Amery WK, Waelkens J. Trigger factors in migraine: a study conducted by the Belgian Migraine Society. Headache. 1987;27(4):191–6. 10.1111/j.1526-4610.1987.hed2704191.x. PubMed PMID: 3597073. 3597073 10.1111/j.1526-4610.1987.hed2704191.x

[9] Karapinar U, SaĞLam Ö, Altundag A, Coskun N, Çetin B, Dursun E. The Role of Lifestyle Modifications in the Management of Migraine Associated Vertigo. Journal of Clinical and Analytical Medicine. 2015;6(6):763–5.

[10] Rist PM, Buring JE, Kurth T. Dietary patterns according to headache and migraine status: a cross-sectional study. Cephalalgia. 2015;35(9):767–75. 10.1177/0333102414560634. PubMed PMID: 25424709; PubMed Central PMCID: PMCPMC4442763. 25424709 10.1177/0333102414560634 PMC4442763

[11] Mirzababaei A, Khorsha F, Togha M, Yekaninejad MS, Okhovat AA, Mirzaei K. Associations between adherence to dietary approaches to stop hypertension (DASH) diet and migraine headache severity and duration among women. Nutr Neurosci. 2020;23(5):335–42. 10.1080/1028415x.2018.1503848. PubMed PMID: 30064351. 30064351 10.1080/1028415X.2018.1503848

[12] Matthews SB, Waud JP, Roberts AG, Campbell AK. Systemic lactose intolerance: a new perspective on an old problem. Postgrad Med J. 2005;81(953):167–73. 10.1136/pgmj.2004.025551. PubMed PMID: 15749792; PubMed Central PMCID: PMCPMC1743216. 15749792 10.1136/pgmj.2004.025551 PMC1743216

[13] Pasta A, Formisano E, Calabrese F, Plaz Torres MC, Bodini G, Marabotto E, Pisciotta L, Giannini EG, Furnari M. Food Allergies and IBS: Lights and Shadows. Nutrients. 2024;16(2):265. PubMed PMID: 10.3390/nu16020265. PMID: 38257158; PMCID: PMC10821155. 10.3390/nu16020265 PMC10821155 38257158

[14] Heizer WD, Southern S, McGovern S. The role of diet in symptoms of irritable bowel syndrome in adults: a narrative review. J Am Diet Assoc. 2009;109(7):1204–14. 10.1016/j.jada.2009.04.012. PubMed PMID: 19559137. 19559137 10.1016/j.jada.2009.04.012

[15] Finkel AG, Yerry JA, Mann JD. Dietary considerations in migraine management: does a consistent diet improve migraine? Curr Pain Headache Rep. 2013;17(11):373. 10.1007/s11916-013-0373-4. PubMed PMID: 24068338. 24068338 10.1007/s11916-013-0373-4

[16] Martin VT, Vij B. Diet and headache: Part 1. Headache: J Head Face Pain. 2016;56(9):1543–52. 10.1111/head.12953. 10.1111/head.12953 27699780

[17] Blackwell B, Mabbitt LA. Tyramine in cheese related to hypertensive crises after monoamine-oxidase inhibition. Lancet. 1965;1(7392):938–40. 10.1016/s0140-6736(65)91257-2. PubMed PMID: 14275713. 14275713 10.1016/s0140-6736(65)91257-2

[18] D’Andrea G, Terrazzino S, Fortin D, Cocco P, Balbi T, Leon A. Elusive amines and primary headaches: historical background and prospectives. Neurol Sci. 2003;24(Suppl 2):S65–7. 10.1007/s100720300044. PubMed PMID: 12811595. 12811595 10.1007/s100720300044

[19] D’Andrea G, Gucciardi A, Leon A. Elusive amines: migraine depends on biochemical abnormalities. Neurol Sci. 2022;43(11):6299–304. 10.1007/s10072-022-06241-2. PubMed PMID: 35840874. 35840874 10.1007/s10072-022-06241-2

[20] D’Andrea G, D’Amico D, Bussone G, Bolner A, Aguggia M, Saracco MG, et al. The role of tyrosine metabolism in the pathogenesis of chronic migraine. Cephalalgia. 2013;33(11):932–7. 10.1177/0333102413480755. 23493762 10.1177/0333102413480755

[21] Vuralli D, Ceren Akgor M, Gok Dagidir H, Gulbahar O, Yalinay M, Bolay H. Lipopolysaccharide, VE-cadherin, HMGB1, and HIF-1α levels are elevated in the systemic circulation in chronic migraine patients with medication overuse headache: evidence of leaky gut and inflammation. J Headache Pain. 2024;25(1):23. 10.1186/s10194-024-01730-5. PubMed PMID: 38369488; PubMed Central PMCID: PMCPMC10875763. **This article reviews the mechanism of food triggers in migraine and medication overuse headache in human**. 38369488 10.1186/s10194-024-01730-5 PMC10875763

[22] Fila M, Chojnacki C, Chojnacki J, Blasiak J. Is an "Epigenetic Diet" for migraines justified? The case of folate and DNA methylation. Nutrients. 2019;11(11). 10.3390/nu11112763. PubMed PMID: 31739474; PubMed Central PMCID: PMCPMC6893742. 10.3390/nu11112763 PMC6893742 31739474

[23] Furlong TJ, DeSimone J, Sicherer SH. Peanut and tree nut allergic reactions in restaurants and other food establishments. J Allergy Clin Immunol. 2001;108(5):867–70. 10.1067/mai.2001.119157. PubMed PMID: 11692117. 11692117 10.1067/mai.2001.119157

[24] Izquierdo-Casas J, Comas-Basté O, Latorre-Moratalla ML, Lorente-Gascón M, Duelo A, Soler-Singla L, et al. Diamine oxidase (DAO) supplement reduces headache in episodic migraine patients with DAO deficiency: A randomized double-blind trial. Clin Nutr. 2019;38(1):152–8. 10.1016/j.clnu.2018.01.013. PubMed PMID: 29475774. 29475774 10.1016/j.clnu.2018.01.013

[25] Ferrara LA, Pacioni D, Di Fronzo V, Russo BF, Speranza E, Carlino V, et al. Low-lipid diet reduces frequency and severity of acute migraine attacks. Nutr Metab Cardiovasc Dis. 2015;25(4):370–5. 10.1016/j.numecd.2014.12.006. PubMed PMID: 25698152. 25698152 10.1016/j.numecd.2014.12.006

[26] Nettleton JA, Steffen LM, Schulze MB, Jenny NS, Barr RG, Bertoni AG, et al. Associations between markers of subclinical atherosclerosis and dietary patterns derived by principal components analysis and reduced rank regression in the Multi-Ethnic Study of Atherosclerosis (MESA). Am J Clin Nutr. 2007;85(6):1615–25. 10.1093/ajcn/85.6.1615. PubMed PMID: 17556701; PubMed Central PMCID: PMCPMC2858465. 17556701 10.1093/ajcn/85.6.1615 PMC2858465

[27] Szilagyi A, Ishayek N. Lactose intolerance, dairy avoidance, and treatment options. Nutrients. 2018;10(12). 10.3390/nu10121994. PubMed PMID: 30558337; PubMed Central PMCID: PMCPMC6316316. 10.3390/nu10121994 PMC6316316 30558337

[28] Vernia P, Di Camillo M, Marinaro V. Lactose malabsorption, irritable bowel syndrome and self-reported milk intolerance. Dig Liver Dis. 2001;33(3):234–9. 10.1016/s1590-8658(01)80713-1. PubMed PMID: 11407668. 11407668 10.1016/s1590-8658(01)80713-1

[29] Evans EW, Lipton RB, Peterlin BL, Raynor HA, Thomas JG, O’Leary KC, et al. Dietary intake patterns and diet quality in a nationally representative sample of women with and without severe headache or migraine. Headache. 2015;55(4):550–61. 10.1111/head.12527. PubMed PMID: 25758250; PubMed Central PMCID: PMCPMC4443434. 25758250 10.1111/head.12527 PMC4443434

[30] Aydinlar EI, Dikmen PY, Tiftikci A, Saruc M, Aksu M, Gunsoy HG, et al. IgG-based elimination diet in migraine plus irritable bowel syndrome. Headache. 2013;53(3):514–25. 10.1111/j.1526-4610.2012.02296.x. PubMed PMID: 23216231. 23216231 10.1111/j.1526-4610.2012.02296.x

[31] Özön A, Karadaş Ö, Özge A. Efficacy of diet restriction on migraines. Noro Psikiyatr Ars. 2018;55(3):233–7. 10.5152/npa.2016.15961. PubMed PMID: 30224869; PubMed Central PMCID: PMCPMC6138234. 30224869 10.5152/npa.2016.15961 PMC6138234

[32] Alpay K, Ertas M, Orhan EK, Ustay DK, Lieners C, Baykan B. Diet restriction in migraine, based on IgG against foods: a clinical double-blind, randomised, cross-over trial. Cephalalgia. 2010;30(7):829–37. 10.1177/0333102410361404. PubMed PMID: 20647174; PubMed Central PMCID: PMCPMC2899772. 20647174 10.1177/0333102410361404 PMC2899772

